# Antifactor Xa activity in critically ill patients receiving antithrombotic prophylaxis with standard dosages of certoparin: a prospective, clinical study

**DOI:** 10.1186/cc3792

**Published:** 2005-08-09

**Authors:** Stefan Jochberger, Viktoria Mayr, Günter Luckner, Dietmar R Fries, Andreas J Mayr, Barbara E Friesenecker, Ingo Lorenz, Walter R Hasibeder, Hanno Ulmer, Wolfgang Schobersberger, Martin W Dünser

**Affiliations:** 1 Department of Anesthesiology and Critical Care Medicine, Innsbruck Medical University, Innsbruck, Austria; 2 Professor, Department of Anesthesiology and Critical Care Medicine, Innsbruck Medical University, Innsbruck, Austria; 3 Professor, Department of Anesthesiology and Critical Care Medicine, Krankenhaus der Barmherzigen Schwestern, Ried im Innkreis, Austria; 4 Professor, Institute of Medical Biostatistics, Innsbruck Medical University, Innsbruck, Austria; 5 Professor, Institute for Leisure, Travel and Alpine Medicine, University for Health Sciences, Medical Informatics and Technology, Hall, Austria

## Abstract

**Introduction:**

Deep venous thrombosis with subsequent pulmonary embolism or post-thrombotic syndrome is a feared complication in the intensive care unit. Therefore, routine prophylactic anticoagulation is widely recommended. Aside from unfractionated heparin, low molecular weight heparins, such as certoparin, have become increasingly used for prophylactic anticoagulation in critically ill patients. In this prospective study, we evaluated the potency of 3,000 IU certoparin administered once daily to reach antithrombotic antifactor Xa (aFXa) levels of 0.1 to 0.3 IU/ml in 62 critically ill patients.

**Methods:**

AFXa levels were determined 4, 12 and 24 h after injection of certoparin. Prothrombin time, activated partial thromboplastin time, antithrombin, fibrinogen, hemoglobin, platelet count, serum urea and creatinine concentrations were documented before and 12 and 24 h after injection of certoparin.

**Results:**

Four hours after certoparin injection (n = 32), 28% of patients were within the antithrombotic aFXa range. After 12 and 24 h, 6% achieved antithrombotic aFXa levels. Because of a severe pulmonary embolism in one study patient, an interim analysis was performed, and the dosage of certoparin was increased to 3,000 IU twice daily. This regime attained recommended antithrombotic aFXa levels in 47%, 27%, 40% and 30% of patients at 4, 12, 16 and 24 h, respectively, after twice daily certoparin injection (n = 30). Antithrombin and fibrinogen concentrations slightly increased during the observation period. Low antithrombin concentrations before certoparin were independently correlated with underdosing of certoparin. Patients with aFXa levels <0.1 IU/ml 4 h after certoparin injection required vasopressors more often and had lower serum concentrations of creatinine and urea than patients with antithrombotic aFXa levels.

**Conclusion:**

Standard dosages of certoparin of 3,000 IU given once or twice daily are ineffective for attaining the recommended aFXa levels of 0.1 to 0.3 IU/ml in critically ill patients. Low antithrombin levels before certoparin administration were independently associated with low aFXa levels. Renal function and vasopressor therapy may further influence the effectiveness of certoparin in ensuring adequate antithrombotic prophylaxis.

## Introduction

Deep venous thrombosis is a feared complication in the intensive care unit, occurring in up to one-third of patients without prophylactic anticoagulation [1]. Pulmonary embolism and/or post-thrombotic syndrome may significantly increase morbidity and mortality in the acute and/or chronic setting of thromboembolic complications [2,3]. Risk factors for the development of deep venous thrombosis in critically ill patients include high age and trauma, heart failure or central nervous system injury as admission diagnoses. Further factors contributing to the development of thromboembolic complications are immobilization, reduction of muscle tone due to analgosedation or relaxation, mechanical ventilation and vessel injury by catheterization of large vessels [4]. Because the inflammatory network and the coagulation cascade are interconnected [5], patients with the systemic inflammatory response syndrome or sepsis are at high risk of developing venous thrombosis [6,7]. Therefore, prophylactic anticoagulation is generally recommended in all critically ill patients [2,8,9].

Currently, unfractionated heparin is the most widely used drug for antithrombotic prophylaxis in intensive care patients. Short half-life time, low costs and availability of an effective antagonist make it an almost ideal antithrombotic agent [10,11]. Variable pharmacokinetics, irregularities in laboratory monitoring, as well as adverse side effects, including heparin-induced thrombocytopenia, however, have raised concerns about the use of unfractionated heparin [10]. Moreover, current evidence suggests that twice daily low dosage unfractionated heparin (5,000 IU) may not be effective enough to prevent thromboembolism in acutely ill medical patients [12].

These considerations, together with novel pharmacological developments, have led to the increased use of low molecular weight heparins (LMWHs) as alternative antithrombotic agents in critically ill patients. Whereas unfractionated heparin appears to decrease the incidence of deep venous thrombosis by 20%, LMWHs were able to reduce it by another 30% in one study [1]. This additional LMWH-associated benefit in thromboembolic risk reduction was particularly effective in intensive care patients at high risk for thrombotic complications [12]. Accordingly, prophylactic anticoagulation with LMWH has already been suggested to be the preferred strategy in critically ill medical patients [13].

In a recent study, however, our working group observed that standard dosages of the LMWH enoxaparin were ineffective at achieving adequate antithrombotic antiFactor Xa (aFXa) levels in critically ill patients. High body weight and the degree of multiple organ dysfunction syndrome were associated with a high probability of underdosing with enoxaparin [14]. Although certoparin is another frequently used LMWH in the intensive care unit, only limited data exist on its efficacy in preventing thromboembolism in critically ill patients.

Although measurement of aFXa levels has been the most widely used method for assessing LMWH activity and the establishment of a therapeutic range for different LMWHs, results on the relationship between aFXa levels and antithrombotic activity of LMWHs are contradictory [15]. Even if aFXa and anti IIa activities have been well correlated with the dose of subcutaneously injected LMWH [16,17], some human and experimental studies could not demonstrate a strong correlation between antithrombotic activity and *in vitro *aFXa plasma levels [18-20]. In contrast, clinical trials have found a significant statistical relationship between aFXa plasma levels and both thrombotic and haemorrhagic outcomes with different LMWHs [21-24].

In this prospective study, the potency of certoparin in achieving adequate antithrombotic aFXa levels is examined in 62 critically ill patients. Additionally, risk factors associated with inadequate aFXa levels under standard certoparin dosages are evaluated.

## Materials and methods

The present study was performed in a 12 bed general and surgical intensive care unit in a tertiary, university teaching hospital between October 2003 and December 2004. The study protocol was approved by the institutional review board and by the Ethics Committee of the Innsbruck Medical University. Written informed consent was obtained, if possible, from all patients, or otherwise from the closest family members prior to study enrolment.

### Patients

Criteria for study inclusion were indication for thromboembolic prophylaxis, age >19 years, body weight >50 kg and intensive care unit stay ≥3 days. Exclusion criteria were any contraindication for anticoagulation with heparins, treatment with anticoagulatory or antiplatelet drugs, continuous veno-venous hemofiltration or other extracorporal therapies, planned or emergency surgery during the 24 h study period, administration of unfractionated heparin within 24 h preceding study entry, and hemorrhage or hemodilution of >30% of estimated blood volume.

### Study protocol

Three thousand international aFXa units (Ph.Eur. 95–130 IU/mg; aFXa/antiIIa 1.5) of certoparin (= 32 mg) in pre-filled, single-dose syringes (Sandoparin^®^; Sandoz, Kundl, Austria) were administered into the abdominal wall *subcutis *of study patients once daily at 8 a.m. Strict attention was paid to the exact emptying of the whole content of the syringe. AFXa levels, as a measurement of the LMWH's biological activity, were determined on the second day of certoparin administration. Then, aFXa levels were measured before, as well as 4, 12 and 24 h after administration of certoparin.

### AFXa determinations

Blood was collected using 3.13% trisodium citrate containing tubes. In the institutional laboratory, plasma aFXa levels were determined by an amidolytic assay using the specific chromogenic substrate S-2732 and bovine factor Xa as reagents and simultaneous thermal analyzers (Coamatic Heparin^®^; Chromogenix, Milano, Italy). No antithrombin was added to the assay *in vitro*. Test results were expressed in international units per milliliter. The aFXa assay standard calibration curve ranged from 0.1 to 1.3 IU/ml with a minimum limit of quantitation of 0.1 IU/ml. To exclude test-related influences on aFXa results, quality testing of the *in vitro *analysis was performed using dilution series. Thus, it is unlikely that methodological, test-associated errors have altered aFXa measurements in this study.

Three groups of patients were specified according to aFXa levels; AFXa levels of 0.1–0.3 IU/ml were considered to represent effective antithrombotic activity [21]. The first group consisted of patients with aFXa levels <0.1 IU/ml, which we considered to be inadequately anticoagulated according to the predefined antithrombotic range. The second group of patients had aFXa levels within the pre-specified antithrombotic range (0.1 to 0.3 IU/ml). The third group consisted of patients with aFXa levels >0.3 IU/ml.

### Study endpoints

The primary endpoint of this study was to evaluate the potency of 3,000 IU certoparin administered once daily to reach antithrombotic aFXa levels of 0.1–0.3 IU/ml in a critically ill patient population. The secondary study endpoint was to identify independent risk factors for underdosing or overdosing of standard certoparin dosages.

### Measurements and documentation of study parameters

The following data were collected from all study patients at study enrolment: age, sex, body mass index, admission diagnosis, and a modified Goris multiple organ dysfunction score [25] calculated from worst clinical and laboratory parameters on the day of study entry.

Documented laboratory parameters included prothrombin time, activated partial thromboplastin time, antithrombin, fibrinogen, hemoglobin and platelet count. Variables were collected before and 12 and 24 h after the second certoparin administration. Serum urea and creatinine concentrations were determined and reported before and 24 h after study enrolment.

### Statistical analysis

A sample size of 60 patients was precalculated on the basis of a previous prospective study [14]. Shapiro Wilk's tests were used to check for normality distribution of data, which was approximately fulfilled in all parameters except for vasopressor requirements and activated partial thromboplastin time, which were log-transformed. For analysis of demographic, laboratory and clinical data, descriptive statistical methods were applied. Time dependence of laboratory values was analyzed using a nonparametric Friedman ANOVA.

To identify independent risk factors for underdosing or overdosing of certoparin, demographic, laboratory and clinical data were entered into a bivariate correlation model to test for differences between patients within antithrombotic aFXa concentrations versus patients with aFXa levels <0.1 or >0.3 IU/ml 4 h after certoparin injection. In case of significant correlations (*p *< 0.05), variables were entered into a binary logistic regression model to identify independent risk factors. The time point of 4 h after certoparin injection was chosen because antithrombotic activity of LMWH is maximal at 3 to 4 h after subcutaneous injection [21].

For each analysis, a significance level of 5% was applied. All data are given as median values and range, or percentage.

## Results

During the study period, 62 patients were enrolled in the trial. Table 1 presents demographic data of all study patients, as well as admission diagnoses, cardiovascular drug requirements, multiple organ dysfunction syndrome score counts, length of intensive care unit stay, and intensive care unit mortality.

**Figure 1:**
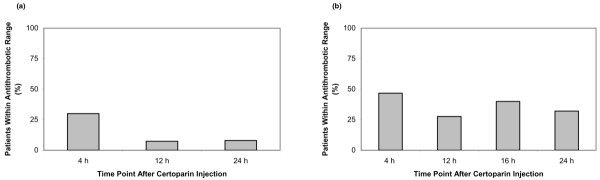
Percentage of patients within recommended antithrombotic range after **(a) **1 × 3,000 IU/d, and **(b) **2 × 3,000 IU/d certoparin.

The percentage of patients within antithrombotic aFXa range at 4, 12 and 24 h after injection of 3,000 IU certoparin once daily (n = 32) is shown in Fig. 1a. Four hours after certoparin administration, median aFXa levels were <0.1 IU/ml (range, <0.1 to 0.2 IU/ml), with 28% (9/32) of patients being within the recommended antithrombotic range of 0.1 to 0.3 IU/ml. Twelve hours after certoparin administration, median aFXa levels were <0.1 IU/ml (range, <0.1 to 0.16 IU/ml), with 6% (2/32) of patients being within the antithrombotic range. Twenty-four hours after certoparin administration, median aFXa levels were <0.1 IU/ml (range, <0.1 to 0.17 IU/ml), with 6% (2/32) of patients being within the antithrombotic range. At no time point did any study patient have aFXa levels >0.3 IU/ml or show clinical signs of bleeding.

Because of a severe pulmonary embolism in one study patient, an interim analysis was performed after inclusion of 32 patients. Following renewed appraisal of the study protocol by the ethical committee, the dosage of the study medication was increased to 3,000 IU twice daily. In this study protocol, 3,000 IU certoparin were administered twice daily at 8 a.m. and 8 p.m. Once again, patients were included into the study protocol only after having received certoparin prophylaxis at 3,000 IU twice a day for one day. AFXa levels were measured before and 4, 12, 16 and 24 h after administration of certoparin. Patients receiving 3,000 IU certoparin once daily were younger than patients receiving 3,000 IU certoparin twice daily. There were no other significant differences between the groups (Table 1).

The percentage of patients within antithrombotic range at 4, 12, 16 and 24 h after injection of 3,000 IU certoparin twice daily is shown in Fig. 1b. Four hours after certoparin administration, median aFXa levels were <0.1 IU/ml (range, <0.1 to 0.28 IU/ml), with 47% (14/30) of patients being within the recommended antithrombotic range. Twelve hours after certoparin administration, median aFXa levels were <0.1 IU/ml (range, <0.1 to 0.26 IU/ml), with 27% (8/30) of patients being within the antithrombotic range. Sixteen hours after the 8 a.m., and four hours after the 8 p.m. certoparin administration, median aFXa levels were <0.1 IU/ml (range, <0.1 to 0.24 IU/ml), with 40% (12/30) of patients being within the antithrombotic range. Twenty-four hours after the 8 a.m., and twelve hours after the 8 p.m. certoparin administration, median aFXa levels were <0.1 IU/ml (range, <0.1 to 0.26 IU/ml), with 30% (9/30) of patients being within the antithrombotic range. At no time point did any study patient develop clinically relevant pulmonary embolism, have aFXa levels >0.3 IU/ml or display clinical signs of bleeding.

Table 2 describes the laboratory results obtained during certoparin therapy in all study patients. During the 24 h observation period, antithrombin and fibrinogen concentrations increased. Although these increases were statistically significant, they occurred in a clinically non-relevant range. There were no changes in prothrombin time, activated partial thromboplastin time, hemoglobin, platelet count, serum creatinine or urea concentrations after certoparin injection. There were no differences in the response of laboratory parameters to 3,000 IU certoparin given once or twice daily.

Table 3 displays bivariate and binary models for identifying independent risk factors for aFXa levels <0.1 IU/ml at 4 h after injection of standard certoparin dosages. In the bivariate analysis, patients with aFXa levels <0.1 IU/ml had significantly lower antithrombin concentrations and higher serum creatinine and urea concentrations, as well as a higher need for vasopressor drugs, than patients within the antithrombotic range. The binary model could identify only low antithrombin concentrations at baseline as an independent risk factor for low aFXa levels 4 h after injection of standard certoparin dosages.

## Discussion

In this prospective study, once and twice daily injection of 3,000 IU certoparin could achieve recommended antithrombotic aFXa levels of 0.1 to 0.3 IU/ml 4 h after administration in only 28% and 47% of patients, respectively. Low antithrombin concentrations before certoparin administration were significantly correlated with low aFXa levels. These results are in striking contrast to earlier studies reporting effective antithrombotic prophylaxis with standard dosages of certoparin (3,000 IU once daily) in high risk patients [26-29].

Despite the fact that certoparin is a frequently used anticoagulant for the prevention of thromboembolic complications in critically ill patients, certoparin proved to be highly ineffective at achieving recommended antithrombotic aFXa levels in this study population. When given at a dosage of 3,000 IU once daily, one patient developed severe pulmonary embolism. In this study patient, certoparin injection could achieve an aFXa level of 0.11 IU/ml 4 h after certoparin administration, whereas aFXa levels were not detectable (<0.1 IU/ml) at 12 and 24 h. Although many individual patient- and critical illness-related factors may have caused pulmonary embolism in this patient, no specific pathogenic factors other than insufficient anticoagulation could be clinically identified.

Similarly, when the dosage frequency of certoparin was increased from 3,000 IU once daily to 3,000 IU twice daily, only 25% to 50% of patients attained antithrombotic aFXa levels during the observation period. It may be speculated that increasing the single dosage from 3,000 IU to 6,000 IU would have resulted in higher aFXa levels during the study period. Whereas a significantly higher proportion of patients would have most likely reached adequate aFXa levels at 4 h after certoparin injection, it is difficult to state whether such an increase in the dosage given once daily would have provided better anticoagulation during the 24 h period than 3,000 IU given twice daily. Moreover, it is currently unknown whether recommended aFXa levels need to be achieved only at 4 h after injection of the LMWH or during the entire dosage interval in order to guarantee adequate antithrombotic protection.

Many pathophysiological mechanisms may have contributed to the observation in this study protocol that aFXa levels were undetectable in the majority of this critically ill patient population. Augmented total body water together with changes in fluid compartments are known to change distribution volume of water soluble drugs like LMWHs in critically ill patients [30]. Furthermore, frequently observed hypoproteinemia and acid-base disturbances can alter the concentration of free, active certoparin in these patients. Aside from such factors, numerous other pathophysiological factors have been reported to influence pharmacokinetics and pharmacodynamics in the intensive care unit patient [31,32]. Low antithrombin concentrations before certoparin injection is one of the most important factors explaining the low aFXa levels after injection of standard certoparin dosages in this study population. LMWHs, as well as unfractionated heparin, exert their anticoagulatory effects by accelerating the inhibitory effects of antithrombin on thrombin formation [33]. Although LMWH-induced bridging between antithrombin and factor Xa is less critical for aFXa activity [34], certoparin could only achieve adequate aFXa levels in this critically ill patient population if antithrombin levels were approximately >70%. Because of ongoing, multifactorial activation of the coagulation system in critical illness, antithrombin levels are often decreased in intensive care patients [35]. Furthermore, patients with low antithrombin concentrations mostly suffer from severe disease and may thus be more likely to receive vasopressor drugs. As indicated in the bivariate statistical model in this study and also in other clinical trials [36,37], patients with cardiovascular failure treated with vasopressor drugs had lower aFXa levels after subcutaneous injection of standard dosages of LMWHs. This is most likely due to reduced subcutaneous blood flow with impaired drug absorption [36].

Because LMWHs are predominantly eliminated by a non-saturable renal mechanism as active or inactive fragments [38], kidney function may substantially influence aFXa levels after certoparin administration. Although it did not reach statistical significance in this multiple regression model, patients with inadequately low aFXa levels after standard certoparin dosages had significantly lower serum creatinine and urea concentrations than patients presenting with antithrombotic aFXa levels between 0.1 and 0.3 IU/ml after certoparin injection. This might be interpreted as better renal function in these patients, which seems to have resulted in a higher clearance of certoparin. Similar effects of renal function on aFXa activity with other LMWHs have been reported [36,39,40].

Interestingly, in a recent prospective trial examining aFXa levels after injection of standard enoxaparin dosages (40 mg once daily) in critically ill patients, our working group observed a significant correlation between aFXa levels and the degree of multiple organ dysfunction syndrome as well as body mass index [14]. In the present study, however, we could not find such a relationship (univariate analysis: body mass index, *p *= 0.322; multiple organ dysfunction syndrome, *p *= 0.988). On the other hand, aFXa levels after enoxaparin injection did not correlate with renal function in the former study [14]. Different pharmacological characteristics of single LMWHs have been reported (e.g. molecular weight distribution, aFXa/antiFactor IIa activity), and could therefore explain differences between enoxaparin and certoparin. When compared with enoxaparin, however, certoparin seems to be inferior for attaining adequate antithrombotic aFXa levels 4 h after injection of standard dosages (56.5% versus 28%, *p *= 0.008; Fisher's exact test).

When interpreting the results of this study, some important limitations must be considered. Assessing the antithrombotic potency of LMWHs by measuring exclusively aFXa activity may underestimate the antithrombotic potency of certoparin by omitting other potential anticoagulatory effects, such as inhibition of thrombin generation, increase of fibrinolytic activity or tissue factor pathway inhibitor, by which LMWH may further influence hemostasis [41]. Therefore, it also cannot be excluded that increasing the absolute dosage of certoparin might result in more bleeding complications. Although no certoparin-associated hemorrhage was observed in this study, the protocol was underpowered with respect to reliably assessing clinical endpoints such as thrombotic or hemorrhagic complications.

## Conclusion

Standard dosages of certoparin of 3,000 IU given once or twice daily are ineffective for attaining recommended antithrombotic aFXa levels of 0.1 to 0.3 IU/ml in critically ill patients. Low antithrombin levels before certoparin administration were independently associated with low aFXa levels 4 h after injection of certoparin. Renal function and vasopressor therapy may further influence the effectiveness of certoparin in ensuring adequate antithrombotic prophylaxis in critically ill patients.

## Key messages

• 	Standard certoparin dosages are ineffective for attaining recommended antithrombotic aFXa levels in critically ill patients.

• 	Low antithrombin levels are associated with low aFXa levels during certoparin prophylaxis.

• 	Renal function and vasopressor therapy may further influence the effectiveness of certoparin.

## Abbreviations

aFXa = antifactor Xa; LMWH = low molecular weight heparin.

## Competing interests

The authors declare that they have no competing interests.

## Authors' contributions

SJ conceived of the study protocol, participated in its design and coordination, carried out bedside sampling and documentation, and helped to draft the manuscript. VM and GL participated in the design of the study and its coordination, and carried out bedside sampling and documentation. DF conceived of the study, helped to perform statistical analysis, and contributed to the draft of the manuscript. AJM conceived of the study protocol and participated in its design and coordination. BEF participated in the study design and its coordination. IL participated in the study design and its coordination. WRH conceived of the study protocol, participated in its design, and helped to draft the manuscript. HU performed the power analysis and the statistical analysis of the data. WS conceived of the study protocol, participated in its design and coordination, and helped to draft the manuscript. MWD conceived of the study protocol, participated in its design and coordination, performed the statistical analysis, and drafted the manuscript. All authors read and approved the final version of the manuscript.

## Figures and Tables

**Table 1:** Demographic data of study patients

	Total	1 × 3,000 IU/d	2 × 3,000 IU/d	*P*-value
n	62	32	30	
Sex (male)	45/62 (72.6%)	20/32 (62.5%)	25/30 (83.3%)	0.09
Age (years)	63 ± 12	60 ± 14	66 ± 10	0.038^a^
BMI	25 ± 4.2	25 ± 5	25 ± 3.3	0.924
Admission diagnoses (n/%)				0.17
Multiple trauma	10/62 (16.1)	5/32 (15.5)	5/30 (16.6)	
Pulmonary disease	4/62 (6.4)	1/32 (3.1)	3/30 (10)	
Cardiac disease	22/62 (35.5)	9/32 (39.1)	13/30 (43.3)	
Infectious disease	5/62 (8.1)	3/32 (9.4)	2/30 (6.6)	
Neoplasm	12/62 (19.4)	9/32 (39.1)	3/30 (10)	
Orthopedic disease	3/62 (4.8)	2/32 (6.3)	1/30 (3.3)	
Other	6/62 (9.7)	3/32 (9.4)	3/30 (10)	
CV drug requirement (n/%)	25/62 (40.3)	13/32 (40.6)	12/30 (40)	1
Dopamine	6/62 (9.7)	5/32 (15.5)	1/30 (3.3)	
Phenylephrine	16/62 (25.8)	10/32 (31.2)	6/30 (20)	
Norepinephrine	7/62 (11.3)	2/32 (6.25)	5/30 (16.6)	
Adrenaline	3/62 (4.8)	1/32 (3.1)	2/30 (6.6)	
Vasopressin	2/62 (3.2)	1/32 (3.1)	1/30 (3.3)	
Milrinone	13/62 (21)	5/32 (15.5)	8/30 (26.6)	
MODS (pts)	4.1 ± 2.1	4.1 ± 2.4	4.2 ± 1.8	0.801
Length of ICU stay (days)	12 ± 11	10 ± 12	13 ± 11	0.301
ICU mortality	2/62 (3.2%)	1/32 (3.1%)	1/30 (3.3%)	1

^a^Significant difference between group 1 × 3,000 IU/d and 2 × 3,000 IU/d. Data are given as mean values ± standard deviation, if not indicated otherwise. BMI, body mass index; CV, cardiovascular; ICU, intensive care unit; MODS, multiple organ dysfunction syndrome.

**Table 2:** Laboratory results during certoparin therapy in all study patients (n = 62)

	Baseline	12 h	24 h	*P*-value
Prothrombin (%)	87 ± 14	88 ± 13	90 ± 14	0.240
aPTT (sec)	39 ± 9	38 ± 7	38 ± 7	0.183
Antithrombin (%)	73 ± 17	75 ± 17	77 ± 17	0.006^a^
Fibrinogen (mg/dl)	497 ± 198	529 ± 201	551 ± 211	<0.001^a^
Hemoglobin (g/dl)	10.2 ± 1.3	10.3 ± 1.3	10 ± 1.1	0.221
Platelets (g/l)	189 ± 103	183 ± 96	199 ± 119	0.055
Serum creatinine (mg/dl)	1.35 ± 0.85	-	1.41 ± 0.95	0.536
Serum urea (mg/dl)	67 ± 40	-	72 ± 42	0.072

^a^, significant time effect.

PT, Prothrombin Time; aPTT, activated Partial Thromboplastin Time; AT, Antithrombin; -, not measured,

Data are given as mean values ± SD.

**Table 3:** Independent risk factors for aFXa <0.1 U/ml at 4 hours after injection of standard certoparin dosages

	Binary model			Bivariate model		
	OR	95% CI	*P*-value	Patients below antithrombotic range	Patients within antithrombotic range	*P*-value

Vasopressor requirement (n/%)	0.348	0.07–1.66	0.185	18/38 (47.4)	5/24 (20.8)	0.038
Antithrombin at baseline (%)	0.910	0.86–0.97	0.002	66 ± 11	86 ± 18	<0.001
Serum creatinine at baseline (mg/dl)	0.302	0.96–1.01	0.302	1.19 ± 0.57	1.7 ± 1.16	0.027
Serum urea at baseline (mg/dl)	0.630	0.17–2.39	0.497	57 ± 34	88 ± 44	0.004

Data are given as mean values ± standard deviation, if not indicated otherwise. aFXa, antiFactor Xa activity; CI, confidence interval; OR, odds ratio.
